# Addressing the Digital Inverse Care Law in the Time of COVID-19: Potential for Digital Technology to Exacerbate or Mitigate Health Inequalities

**DOI:** 10.2196/21726

**Published:** 2021-04-07

**Authors:** Alisha R Davies, Matthew Honeyman, Bob Gann

**Affiliations:** 1 Research and Evaluation Division Public Health Wales Cardiff United Kingdom; 2 Kings Fund London United Kingdom; 3 Digital Communities Wales Cardiff United Kingdom

**Keywords:** COVID-19, digital divide, digital exclusion, digital health, health inequality, population health

## Abstract

Digital technologies have been transforming methods of health care delivery and have been embraced within the health, social, and public response to the COVID-19 pandemic. However, this has directed attention to the “inverse information law” (also called “digital inverse care law”) and digital inequalities, as people who are most in need of support (in particular, older people and those experiencing social deprivation) are often least likely to engage with digital platforms. The response to the COVID-19 pandemic represents a sustained shift to the adoption of digital approaches to working and engaging with populations, which will continue beyond the COVID-19 pandemic. Therefore, it is important to understand the underlying factors contributing to digital inequalities and act immediately to avoid digital inequality contributing to health inequalities in the future. The response to COVID-19 represents a sustained shift to adopting digital approaches to working and engaging with populations which will continue beyond this pandemic. Therefore it is important that we understand the underlying factors contributing to digital inequalities, and act now to protect against digital inequality contributing to health inequalities in the future.

## Introduction

### Background

Technology is transforming the way we live our lives. A digital revolution is underway in the public sector, including the health sector, to facilitate the use of digital solutions to drive efficient systems and improve population health both in the United Kingdom [1-4] and worldwide [5]. The expected benefits to individuals include more rapid access to information and personalized care, more control, and empowerment for their own health. The benefits to health and care systems include opportunities to deliver efficient and effective care closer to patients and target scarce resources in a better manner, using precision medicines, big data, and artificial intelligence.

However, while the application of digital innovation in health care—hereinafter referred to as “digital health”—has gained impetus, the wider social system has received limited consideration, and people most in need of care are also least likely to have access to, or engage with, technology. A failure to acknowledge and address this challenge would imply that the adoption of digital health has the potential to inadvertently widen health inequalities, thus integrating the inverse care law [6] into the digital era as a “digital inverse care law”, previously called “inverse information law” [7].

The COVID-19 pandemic has markedly highlighted the vital role of access to the internet and digital technology in enabling the general public to live their everyday lives during the pandemic. The pace of digital innovation and its adoption in health and care among the general public has accelerated. This includes widespread rapid adoption of internet-based health consultations and the development of digital health tools and apps to protect health and well-being. There has also been a demonstrable increase in the proportion of people in the general population who use the internet to search for information; maintain contact with others; support the continuation of work, study, or home-schooling during the COVID-19 pandemic; and access basic needs including shopping and financial support [8,9]. During a period of enforced social isolation, a focus on internet-enabled social responses has implied that people without a presence on the internet are effectively excluded. As we progress towards recovery from the COVID-19 pandemic, there is a risk of leaving behind people who do not engage with digital technologies and are most likely in need of support.

Increasing awareness and improving our understanding of the factors contributing to the digital inverse care law, and how these challenges can be addressed, is of considerable importance in the application of digital health [10,11]. We can expect the increased reliance on digital technologies during the COVID-19 pandemic to be sustained in many ways even after the COVID-19 pandemic; hence, it is important to recognize these issues immediately.

### Objective

To help inform action, here we describe the factors contributing to digital exclusion, its contribution to health and social inequalities, and the potential factors that need to be addressed to prevent it. This viewpoint has been informed by the academic and gray literature on technological advancements in the health sector and seeks to highlight the complexity of the drivers of the digital inverse care law and the actions needed across government, public, private, and nonprofit sector organizations to ensure capitalizing on the potential for digital technologies to address health issues and minimize the risk of the exacerbation of health inequalities.

## Factors Contributing to Digital Exclusion

We describe digital exclusion as a complex challenge that consists of 3 interconnected components, with inequalities evident in each component: (1) access to digital connectivity and infrastructure, (2) digital skills and literacy, and (3) engagement with digital platforms. Here we consider each of these elements, illustrated within the context of the key opportunities for digital technologies in the National Health Service (NHS) in the United Kingdom [11], with particular reference to the COVID-19 pandemic.

### Access to Digital Connectivity and Infrastructure

People worldwide use the internet to stay in touch with others and live their lives during the COVID-19 pandemic. For example, a survey by the Pew Research Center in April 2020 [12] revealed that 53% of people in the United States felt that the internet is essential during the COVID-19 pandemic, and a further 34% felt that it is important but not essential. Nonetheless, only 55% of households worldwide have an internet connection, ranging from 87% of households in high-income countries, 47% in transitional countries, and only 19% in the low-income countries [13].

Even in high-income countries, marked inequalities in internet access are evident. In 2019, 7% of households in the United Kingdom did not use the internet [14], and 10% of adults in the United Kingdom did not use the internet regularly [15]. The same proportion has been reported in the United States, increasing with age to 27% among people aged ≥65 years [16]. A population-wide survey across 17 European countries revealed that 51% of people aged ≥50 years do not use the internet [17].

Internet access can be facilitated by enabling access to internet-enabled technologies alongside a fixed (eg, household) or mobile broadband connection. While internet access through a fixed broadband connection has increased in recent years, the quality and speed of the connection remains poor for many people, and this is particularly the case in rural areas. Data from the European Union suggest that the increase in very-high-capacity network coverage in rural areas remains significantly lower than the total coverage, despite marked improvements in recent years (from 2011 to 2019, very-high-capacity network coverage increased from 2% to 20% in rural areas, compared to an increase from 10% to 44% overall) [18]. In the United Kingdom, residential premises in rural areas have lower coverage of fixed superfast broadband connections (79% of properties in rural areas compared to 97% in urban areas), download speeds exceeding 10 Mbps (83% in rural areas compared to 98% in urban areas), and lower access to high-quality mobile data (4G data services; 41% in rural areas compared to 85% in urban areas) [19]. Regional disparities in broadband access are not a problem exclusive to rural areas. The National Digital Inclusion Alliance’s report on the worst connected cities revealed that among 221 large- and medium-sized US cities, at least 30% of households lacked a broadband connection [20].

A lack of rapid, reliable connectivity across fixed and mobile internet services can be a challenge for patients to receive remote care and for health care staff who are mobile and work in patients’ homes. In a survey among the members of the Queen’s Nursing Institute (n=534) [21], 454 (85%) nursing professionals reported that poor connectivity in patients’ homes is the greatest challenge to effective mobile working in the community.

Alongside infrastructure, another factor contributing to internet access is the affordability of internet-enabled digital devices and data plans. Over 25,000,000 mobile phone users in the United Kingdom are pay-as-you-go customers, with the majority of users having a low income. Community organizations have reported examples of vulnerable groups spending up to half the family budget on incurring mobile phone costs [22]. Described as “data poverty,” accessing the internet through mobile digital technologies can be an unaffordable essential need and individuals may be reluctant to devote scare data resources to digital health in light of competing demands [23].

The COVID-19 pandemic response has exposed digital poverty with the lack of ownership of digital devices and low affordability of data plans [24]. In addressing these challenges, the UK Government supported the DevicesDotNow initiative, which asked businesses to donate devices (tablets, smartphones, and laptops) and connectivity (in the form of SIM cards, dongles, and mobile hotspots) to be distributed to households who would otherwise be digitally excluded [25]. In Wales, there has been a rapid roll out of the Attend Anywhere video consultation service, accompanied by the supply of 1000 tablet devices to hospitals, care homes, and hospice settings, to enable vulnerable people to access the service on the internet [26]. In addressing data affordability, telecommunication companies in the United Kingdom have removed the data cap for fixed broadband contracts during the COVID-19 pandemic [27]. However, this did not apply to pay-as-you-go mobile contract holders; therefore, those likely to be in greater need were not able to benefit from this initiative.

Evaluation of the impact of such initiatives would help understand the extent to which such programs reach people who are most in need and address the access and affordability drivers of digital inequalities across all groups.

### Digital Skills and Literacy

In 2020, a survey in the United Kingdom revealed that 10,500,000 people (16% of the adult population of the United Kingdom) cannot perform basic activities with digital devices, such as turning on a device, connecting to the Wi-Fi, or opening an app by themselves. In total, 7% of the population of the United Kingdom (3,600,000 people) is almost completely offline [28]. Data from the Digital Economy and Society Index of the European Commission suggest that while the level of digital skills continues to increase across many countries in recent years, progress among different population groups is highly variable. In 2019, 82% of young people (aged 16-24 years) and 85% of those with high formal education have at least basic digital skills, compared to only 35% of people aged 55-74 years [29].

There are a number of specific programs in the United Kingdom [30-32] and worldwide [33,34], which seek to address gaps in digital skills among specific groups of people. Structured programs, such as those provided by the Good Things Foundation [35] and Digital Communities Wales [36], focus on overcoming the barriers to opportunity, access, knowledge, and skills for using technology (particularly the internet). During the COVID-19 pandemic, web-based digital skills programs for the general public, such as Learn My Way [37], have been made available free of charge, thus eliminating financial barriers to internet access. Many other initiatives supporting the best practices in digital skills development across Europe are highlighted in the European Commission annual Digital Skills Awards [38], including, for example, community navigators to help older people living with long-term conditions to access and use technologies [39]. Digital skills programs tend to be focused on the general public; however, there is also the need to develop the digital skills among the NHS workforce, such that they can support patients to engage with a digital health care system and direct them to effective digital solutions. In a study on effective mobile working in the community, 21% of nurses reported that limited or no training for the use of the devices was a key challenge to implementation [21]. Examples of nationwide programs supporting digital skills training among health professionals include the Digital Readiness program of Health Education England [40].

Globally, digital literacy and continuous skill development as technologies evolve has been identified as an important driver of health technology use [5]. Ensuring everyone is equipped with the digital skills needed to effectively engage with digital technology is crucial to address digital exclusion.

### Engagement With Digital Platforms

It is important to address internet access and digital skill gaps, but these factors alone are unlikely to be sufficient to ensure all patients have the potential to benefit from services delivered on digital platforms. Many other factors potentially influence an individual’s choice to engage with digital platforms; these include levels of awareness, trust, and perceived benefits, the combination of which would differ across age groups, genders, and socioeconomic and cultural backgrounds.

As evidenced by the latest issue of the Consumer Digital Index of the United Kingdom [28], a lack of motivation or interest is one of the key barriers to internet engagement. Over one-third of internet nonusers claim that the internet does not interest them, and 48% claim that “nothing” could motivate them to become internet users. Alongside motivation, there is also a need to consider that different groups of people prefer to engage with different digital platforms. A survey conducted in the United Kingdom reported that although over 50% of adults were willing to have web-based consultations with general practitioners, approximately 25% of people aged over 65 years and 40%-45% of people from households earning less than £25,000 would not opt for a web-based video consultation with their general practitioner [11]. The COVID-19 pandemic has forced many primary care consultations to be carried out remotely through the telephone and web-based or video platforms, leading to widespread implementation of these forms of consultation as practical solutions [41]. Some studies have suggested high satisfaction among people who engage with online care [42]; nonetheless, few studies have evaluated the levels of engagement across different populations, the underlying contributing factors, and the impact on timely access to health information, care, patient experience, and health outcomes. More comprehensive evaluation is needed to understand and inform the development of digital or mixed models of service in a better manner to ensure that digital innovation does not inadvertently contribute to the exacerbation of inequalities in outcomes.

Coproduction is essential to address disparities in the engagement with digitally delivered health care among diverse populations. Tools such as the “Culturally-Informed Design Framework” in the United States are useful guides to encourage developers and providers to consider cultural differences in engagement to optimize the choice of digital platform, functionality, content, and user interface [43].

## How Digital Exclusion May Exacerbate Health and Social Inequalities

The digital era has transformed and continues to transform society rapidly, influencing our access to information, employment, working conditions, health and care services, and social connections, and in turn the conditions for good health. However, alongside advancements in digital innovation, digital exclusion is increasingly being recognized as an important factor potentially contributing to both health and social inequalities [44].

The evidence summarized here describes how digital exclusion is a complex factor that reflects underlying social and economic inequalities (such as economic barriers to accessing data and devices or variations in regional internet infrastructure) and contributes to social, economic, and health inequalities (as those less able or likely to engage with digital technology are also most likely to need support).

**Figure 1:**
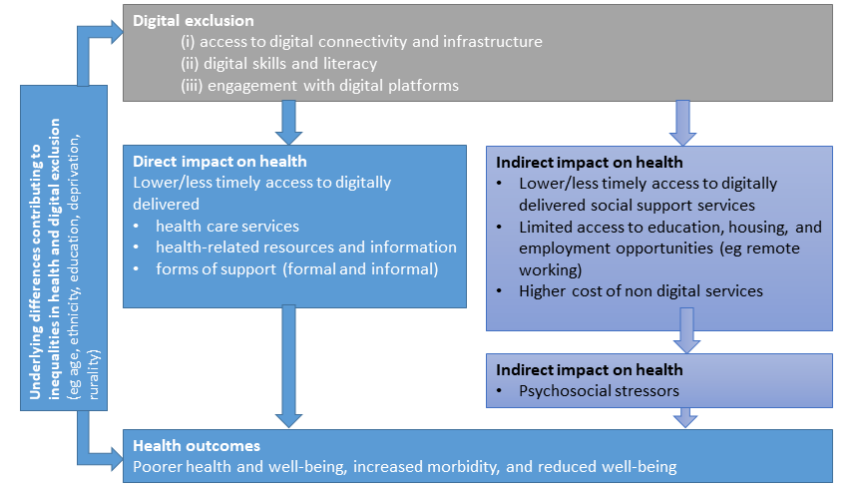
The direct and indirect impacts of digital exclusion on health inequalities. Adapted from McAuley [45].

The digital inverse care law [7,45] reflects the direct impact of digital exclusion on health, for example by reducing an individual’s access to timely and reliable health information, services, and support (eg, from professionals or peer support groups) delivered on digital platforms (Figure 1). A focus on digitally delivered health systems, without due consideration of digital exclusion, has the potential to prevent “intervention generated inequalities” from reinforcing the underlying inequalities in health [46,47]. In a nationwide representative study of engagement with digital technology for health purposes in Wales, the use of digital technology was lower among groups of people who are likely to have greater health needs, including older people, those living in less affluent areas, those with poorer underlying health, and those reporting health-harming behaviors (ie, smoking, drinking, and physical inactivity) [48]. This has also been highlighted within the context of COVID-19, a period when having an internet presence is crucial to rapidly access not only health information but also digital health consultations and health monitoring apps. Furthermore, people who are at the highest risk of poor health outcomes are also those most likely to be digitally excluded, including older people and those living in more deprived areas [49].

Digital exclusion can also have an indirect impact on health outcomes, acting through the other social determinants of health [44]. For example, digital exclusion may reduce an individual’s access to social support services (eg, income support), education, and employment opportunities, all of which are recognized as underlying social and economic determinants of health (Figure 1). Within the context of the COVID-19 pandemic, during the first 2 weeks of the nationwide lockdown in the United Kingdom, approximately 1,000,000 people sought financial support through universal credit; however, this was only possible on the internet, thus posing a challenge to those who are digitally excluded. When schools, crèches, universities, offices, churches, shops, restaurants, and parks are closed owing to the COVID-19 pandemic, the usual institutions around which people structure their everyday lives and gain support were no longer physically accessible. Many people transitioned to web-based platforms, which was a supportive community inaccessible to digitally excluded people [50].

Digital exclusion is concurrent with Dahlgren and Whitehead’s definition of a social determinant of health [51], as a social and economic factor with the potential to increase or decrease social inequities in health. Recognition of digital exclusion as a social determinant of health by governments and systems would increase the visibility of the issue, highlight the need for routine measurement, report variations across population groups, and focus action on the factors contributing to digital exclusion across policy, health, and social systems. There are examples where health systems display different behaviors in light of increased awareness of digital exclusion, such as addressing data poverty by enabling mobile phone users toll-free access to the NHS and national government health websites [52]. Digitally excluded people would have been hugely impacted without access to rapidly changing and reliable public health advice during the COVID-19 pandemic.

## Digital Innovation, COVID-19, and Health Inequalities: an Example

In response to the COVID-19 pandemic, contact tracing digital apps were rapidly implemented in several countries [53] including the United Kingdom [54]. Engagement with the app is dependent on the individual owning a smartphone, having the skills to understand and download the app, and keeping their phone switched on with Bluetooth enabled. However, 6,500,000 adults in the United Kingdom cannot turn on a device, and 5,900,000 adults cannot open an app [28]. A survey carried out by IPSOS Mori for the Health Foundation [55] revealed a clear digital divide by age, occupation, and educational level in public readiness to download the NHS COVID-19 app. Public trust and confidence were also likely to be highly instrumental in adoption. One’s motivation to download and use the NHS COVID-19 app would reflect one’s views on risk and benefit within the context of a pandemic and, potentially influenced by the views of one’s family, social networks, and professionals.

Contact tracing apps are likely to be disproportionately taken up by younger, more affluent, and tech savvy populations. The benefit of the app extends to users and people around them; however, if digital exclusion prevents the most vulnerable people from participating in digital technologies, then the collective societal benefits are not equitable. This highlights the importance of digital innovation being accompanied by nondigital approaches, which are efficient and effective for people who cannot or choose not to engage with digital solutions, and to evaluate the outcome across population groups [56].

## Actions to Mitigate the Risk of Digital Inequalities Exacerbating Health Inequalities

There is sufficient evidence to conclude that digital exclusion should be considered across sectors as a social determinant of health, with the potential to exacerbate health inequalities if progress does not consider the structural, economic, social, and behavioral factors contributing to digital exclusion.

The COVID-19 pandemic has clearly highlighted the levels of inequality in the ownership of digital devices and demonstrated that the access to fixed and mobile internet connections is a gateway to essential health information and care and many other key services including education, food delivery, employment, and social support, all of which indirectly impact health. As described here, the underlying inequalities in the access to digital technology and the internet are evident, and if left unresolved, have the potential to exacerbate health and social inequalities in a digital era.

In order to progress towards digital inclusion, one policy approach adopted by certain countries is to declare that access to the internet is a human right [57], whereas others consider technology and the internet as an enabler of rights rather than a right in itself [58]. Declaration of access to the internet as a right may enforce action at national and international levels to address a factor that indirectly contributes to health, placing it at par with other rights including the access to food and safe housing, all of which contribute to health equality.

This study shows that the underlying drivers of digital exclusion are complex, and as such the solution cannot be obtained through a single action or from a single organization. Addressing digital exclusion would require government, public, private, and nonprofit sector organizations to collaborate to ensure that progress toward a digital future does not inadvertently leave some people behind. This will require a comprehensive understanding of the factors contributing to digital exclusion in local populations and how these factors differ among groups, areas, and over time, along with effective engagement with digitally excluded people to coproduce solutions that adequately address the contributing factors such as poverty, access, skills, or motivation.

For example, a common viewpoint is that addressing barriers to internet connectivity will address digital exclusion; however, this is not the case. Inequalities would continue to persist after access is resolved if the complex underlying factors that contribute to the digital divide are not addressed. a crucial first step is to understand the extent and drivers of digital exclusion within and between different populations through data and qualitative insights. Followed by a multidisciplinary and multisectoral response to address structural barriers (such as the lack of infrastructure to support adequate internet access in all areas), financial barriers (eg, costs of devices and data plans), digital skills and literacy, and other barriers to engagement (eg, cultural, concerns regarding trust and data privacy) (Table 1). Embedded evaluation alongside digital innovation is essential to ascertain the impact of each of these actions against key outcomes including differences in uptake, engagement and effectiveness across population groups. For progress to occur, there is a need to bring together actions against these key components in a comprehensive and coordinated plan; for example, the New York City Internet Masterplan [59] is a comprehensive roadmap to close the digital divide through enhanced infrastructure, affordability, and inclusion. To ensure collective progress, it may also be necessary to ensure that the government takes the responsibility for digital equity.

Organizations in the United Kingdom have brought together practical calls to action to mitigate the risk of digital inequalities. The Good Things Foundation has issued a blueprint for a 100% digitally included United Kingdom for a post–COVID-19 economy, focusing on 3 key steps to fix the digital divide: the need to address the digital infrastructure, data poverty, and to develop an inclusive digital strategy [60]. The Carnegie UK Trust has reported 12 recommendations for policymakers, practitioners, academics, and industry professionals to learn from the lockdown and eliminate digital exclusion [61]. In Wales, the Older People’s Commissioner has urged investment in digital inclusion as one of the preconditions for an age-friendly recovery [62]. With an international focus, particularly on low-income countries, the Human Rights, Big Data and Technology Project has postulated 5 urgent principles for leaving nobody behind through technology in the COVID-19 response [63]: guaranteeing internet access as a human right and a public good, increasing the availability and acceptability of the digital infrastructure, increasing the accessibility and affordability of digital services, empowering people by addressing disinformation and hate speech without censorship, and ensuring that internet access is not a cause for more surveillance.

**Table 1:** Data and insights on the extent and drivers of digital exclusion leading to digital inequalities in population subgroups and potential approaches to address inequalities and to evaluate and understand the uptake and effectiveness of digital technologies and differences across population subgroups.

Driver of digital inequality	Types of digital inequalities	Suggested approaches to address inequalities
Structural barriers (access)	Inequalities in access to a rapid and reliable internet connection	Infrastructure investment Report publicly available data on internet coverage across populations
Financial barriers	Inequalities in the affordability of internet-enabled digital technology and data plans	Understand economic barriers including internet access, affordability of technology, data poverty through research, and engagement with service users Examine the impact of different policies and economic responses (eg, financial support, waived data charges for essential services, subsided costs, market regulations to reduce cost, and data caps)
Digital skills and literacy	Inequalities in digital skills among service users	Nationwide and local systems should use existing measures of digital inclusion to understand population needs, such as the general Digital Inclusion Scale [64] or the health-specific eHealth Literacy Scale [65] Implement structured programs to address skills gaps such as the NHS Widening Digital Participation delivered of the Good Things Foundation [35,37] and Digital Communities Wales [36] Targeted support addressing essential digital skills among vulnerable groups (eg, through peer-to-peer support, intergenerational mentoring, and localized digital champions [66])
	Inequalities in digital skills, knowledge, understanding, and awareness among service providers and the workforce	Develop a model of digital competence across different roles, train and support the workforce to enable a digital future (eg, the NHS Digital Readiness program [40])
Engagement with digital platforms	Differences in motivation, trust, and perception of risks across populations	Develop a nationwide standard instrument routinely administered to users to understand factors contributing to engagement Coproduce digital solutions tailored to service user needs and levels of digital skills or engagement. Address barriers of trust and risk with transparency and clarity on the collected data and the underlying purpose
Design and development of services	Incompatibility across different platforms (fixed or mobile) Inoperability owing to lower internet speeds Requiring the use of data plans	Include digital inequality as a factor in the equality impact assessments alongside service development and delivery Incentivize partnerships across public, private, and nonprofit sector organizations to address digital exclusion Ensure new service developments considering digital exclusion Contracts with suppliers to include reports on granular data on service use in local health systems across platforms to identify excluded populations

As health and care systems seek to deliver services through digital platforms, it remains important to monitor and evaluate the levels of engagement to ensure that a focus on digital approaches does not inadvertently reinforce underlying inequalities in health. Within the United Kingdom, the NHS provides a potential opportunity to monitor and address different levels of engagement with digital technology to help understand differences by clinical needs and population groups, an understanding of which could be shared to inform innovation elsewhere.

Based on theoretical frameworks and the lessons learned from the practical examples described here, the actions needed to ensure that digital health innovation helps address, rather than exacerbate, health inequalities are as follows:

1. Understand the extent and drivers of digital exclusion in population subgroups
2. Use existing measures or develop new measures of digital exclusion and digital health literacy
3. Ensure that digital strategies and new service developments consider factors contributing to local exclusion
4. Develop solutions through coproduction for a maximally extensive and appropriate user base
5. Where widespread adoption is required, ensure the technologies used have the characteristics of innovations capable of being rapidly disseminated [67-69]
6. Use realistic and evidence-based models of behavioral science to inform digital health innovation, such as the COM-B system [70].

## Conclusion

Digital technology has the potential to revolutionize health and health care with growing interest among policymakers, researchers, and practitioners in exploring how digital technology can be harnessed to improve population health. Countries worldwide have been using digital technologies to respond to the COVID-19 pandemic, for applications including communication of public information, remote delivery of health care, and population surveillance [71]. Greater adoption of technology can also provide additional sources of valuable data for potential use by population health systems to understand health needs more clearly, monitor outcomes, and counter the factors underlying ill health.

Digital innovations should be accessible to everyone, empowering citizens to become active contributors to health and well-being. However, those likely to have the greatest health needs are also least likely to have access to digital platforms and the skills to use and navigate them, and they are hence less likely to engage with such digital platforms. This raises an important question of equity in population health in a digital era, an issue brought into sharp focus by the COVID-19 pandemic. This led us to ask how we can innovate through digital technology and transform population health, while leaving no one behind.

To address this question, we need to better understand who engages with digital technologies, the enablers and barriers, and the direct and indirect impact on health outcomes. The lack of consideration of these factors poses the danger that the pursuit of digital health solutions results in unintended consequences and reinforces existing social and health inequalities. Action is needed across government, public, private, and nonprofit sector organizations to ensure capitalization on the potential for digital technology to address health and minimize the risk of exacerbation of health inequalities.

## Abbreviations

NHS: National Health Service
